# Rapid detection of Atlantic salmon multi‐quality based on impedance properties

**DOI:** 10.1002/fsn3.1362

**Published:** 2020-01-21

**Authors:** Zongbao Sun, Liming Liang, Junkui Li, Xiaoyu Liu, Jian Sun, Xiaobo Zou, Min Zuo, Zhiming Guo

**Affiliations:** ^1^ School of Food and Biological Engineering Jiangsu University Zhenjiang China; ^2^ School of Electrical and Information Engineering Jiangsu University Zhenjiang China; ^3^ National Engineering Laboratory for Agri‐product Quality Traceability Beijing Technology and Business University Beijing China

**Keywords:** Atlantic salmon, chemometrics, impedance properties, multi‐quality, rapid detection

## Abstract

To establish a rapid, convenient, and low‐cost method to assess the quality of Atlantic salmon, we analyzed the impedance between 10^–1^ and 10^5^ Hz for Atlantic salmon/rainbow trout, chilled/frozen‐thawed salmon, and fresh/stale salmon. We combined chemometrics with impedance properties to create a multi‐quality index for Atlantic salmon. The accuracy of all three models established can reach 100% in distinguishing Atlantic salmon from rainbow trout and distinguishing chilled salmon from frozen‐thawed salmon. We applied a partial least squares method to create a quantitative prediction model of bioimpedance spectroscopy and the value of total volatile basic nitrogen. The correlation coefficients of the training and test sets were 0.9447 and 0.9387. Our results showed that the combination of impedance properties and chemometrics was a simple and effective application to evaluate Atlantic salmon quality.

## Graphical abstract

We established a multi‐quality rapid detection method to detect different quality Atlantic salmon by combining chemometrics with impedance properties. And the characteristics of bioimpedance spectra of salmon with different qualities were analyzed theoretically.

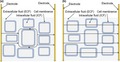

## 1 INTRODUCTION

Atlantic salmon is rich in many valuable nutrients, such as high‐quality polyunsaturated fats, vitamins (tocopherols), and carotenoid pigments (astaxanthin; Johnston et al., 2006). Thus, Atlantic salmon has long been one of the most popular of all kinds of seafood. However, because of a lack of effective means of detection, fraud in the Atlantic salmon consumer market appears frequently, mainly in the following ways: (a) Low‐priced rainbow trout is marketed as high‐priced Atlantic salmon; (b) low‐priced rainbow trout is marketed as high‐priced Atlantic salmon; (c) stale salmon is sold as fresh salmon.

Consumers expect that the salmon they buy is quality‐guaranteed. Many studies have been performed on detection of the quality of Atlantic salmon and salmon products to satisfy consumer expectations, protect the legitimate rights and interests of consumers, and to standardize Atlantic salmon markets. These studies have included physical and chemical detection (Aursand, Erikson, & Veliyulin, 2010; Rodríguez et al., 2012), sensory evaluation (Quevedo & Aguilera, 2010; Quevedo, Aguilera, Pedreschi, 2010), and spoilage microorganism analysis (Skipnes, Johnsen, Skåra, Sivertsvik, & Lekang, 2011; Tito, Rodemann, & Powell, 2012). However, most of these methods are destructive, time‐consuming, and inefficient, and they cannot meet the needs of large‐scale commercial applications. Therefore, there is an urgent need for rapid and nondestructive technology to detect and control the quality and safety of Atlantic salmon or Atlantic salmon and its products.

Investigators have used bioimpedance technology to assess meat quality because it is rapid, low‐cost, nondestructive, and portable (Pérez‐Esteve et al., 2014). Zhang, Shen, and Luo (2011) employed impedance characteristics to forecast freshness of grass carp to establish a correlation between impedance measurements and the value of total volatile base nitrogen (TVB‐N), total aerobic count, and sensory assessment. Fuentes et al. (2013) employed impedance spectroscopy to differentiate between unfrozen and frozen‐thawed sea bream. Similarly, Rizo et al. (2013) used an impedance spectroscopy method to analyze the process of salting and smoking of fish products. Additionally, Yang used electrical impedance spectroscopy to develop a rapid detection system for pork moisture content (Yang et al., 2013). Sun et al., (2018) studied the relationship between electrical characteristic and spoilage stages, and he proposed an original fish freshness classification method without a conventional data fitting model. Some researchers have also employed electrical impedance spectroscopy to detect meat component, such as fat, water, and protein content; such work could offer strong theoretical knowledge for the variability of biological impedance spectrum measurement (Bosworth & Wolters 2001; Cox & Hartman 2005; Willis & Hobday 2008).

The foregoing studies notwithstanding, there are few reports on impedance detection of salmon's comprehensive quality, and most studies lack theoretical analysis of bioimpedance characteristics. Accordingly, we analyzed the impedance module and phase angle of Atlantic salmon/rainbow trout, chilled/frozen‐thawed salmon, and fresh/stale salmon. By combining chemometrics with impedance properties, we propose a rapid method for deriving Atlantic salmon multi‐quality indices.

## 2 MATERIALS AND METHODS

### 2.1 Sample collection

Atlantic salmon samples were purchased from Bakkafrost, the largest salmon farming company in the Faroe Islands. The fresh salmon were slaughtered, processed into chilled salmon, and vacuum‐packaged within 3 hr at a processing plant. The chilled salmon were transported to Shanghai Chuner Trade Development Co., Ltd. by cold chain at 4°C and then transported by refrigerated truck to Zhenjiang Metro supermarket. The entire process lasted about 4 days. Rainbow trout samples were purchased from Qinghai Minze Longyang Gorge Ecological Water Reproduction Co. LTD. The trout were slaughtered by the processing plant, vacuum‐packed, and transported to Zhenjiang metro supermarket by cold chain at 4°C for 3 days. Ten fish of both kinds were used in the study.

### 2.2 Sample preprocessing

On arrival in the laboratory, we cut the middle part of the fish into fillets of the same shape (3 × 3 × 2 cm); the heads and tails of the fish were discarded. The fillets were packed in food‐grade vacuum packaging bags (Weide New Material Co., Ltd) and sealed.

First, 48 salmon fillets and 48 rainbow trout fillets were randomly selected for experiments to distinguish fish species. Second, 96 salmon fillets were randomly selected to distinguish between chilled salmon (C‐S) and frozen‐thawed salmon (FT‐S). Half of the 96 salmon fillets were stored at 4 ± 0.5°C (Ali et al., 2015) as C‐S, and half were frozen at 18 ± 0.5°C for 3 days and thawed at 4 ± 0.5°C for 2 days as FT‐S. Then, we analyzed the electrical characteristic of C‐S and FT‐S having the same storage time. Finally, 60 salmon fillets were selected for freshness detection. For prediction of freshness, we considered that the maximum freshness of salmon was the first day when the salmon arrived in the laboratory. Sixty fillets were vacuum‐packed and stored at 4 ± 0.5°C, and, within 12 days, we selected 20 samples randomly every 3 days for freshness detection measurements.

### 2.3 Impedance spectroscopy

The impedance equipment consisted of a computer (CPU AMD E2‐9000, 2.2 GHz, memory 4 G, ASUS), the impedance instrument (CHI660E, CH Instruments, Inc), and an electrode. The sinusoidal voltage of 10^–1^–10^5^ Hz is released by the workstation as the excitation signal. The impedance module and phase angle were calculated automatically by measuring the response current signal. The range of frequencies we selected was according to the model for α and β dispersion (Gheorghiu 1993), and the ability of the electrochemical workstation. A similar range of frequencies was used by other researchers (Chen et al., 2017). In the measurements, we used an electrode (10 mm apart, 1 mm in diameter) composed of four gold‐plated copper needles: Gold is a stable metal that is not damaged by chemical reactions between electrodes and biological tissues. The impedance measurements were made by inserting the electrodes into the fish muscle fibers at an angle of 90° and ensuring that the electrodes were fully introduced into the sample. Fish fillets were placed in small refrigerators and connected to external instruments by wires. The temperature of the fillets was maintained at 4.0 ± 1.5°C, while the impedance measurements were being taken (Pérez‐Esteve et al., 2014). The impedance data were the average of three repeated measurements at the same position of the fish fillets. The data were discarded if the percent of absolute difference in the means among the three measurements was more than 3%.

### 2.4 Physicochemical analysis

The TVB‐N value was assessed by semimicro fixation of nitrogen as described by Sallam (2007) and expressed as mg/100 g of salmon sample. A 10 g sample of salmon muscle was weighed, pulverized, and soaked in 100 ml of aqueous trichloroacetic acid (20 g/L) for 30 min, followed by a filtration step. Next, 5 ml of filtrate and 5 ml of MgO (10 g/L) were distillated using a Kjeldahl apparatus (Huadong Chemical Glass Co., Ltd.), with 5 ml distilled water used as the control. The distillate was collected in 10 ml of aqueous boric acid (20 g/L) with the addition of a mixed indicator consisting of 0.1 g methyl red and 0.1 g of methylene blue in 100 ml of ethanol. The absorption liquid was immediately titrated with a 0.01 mol/L hydrochloric acid solution. The TVB‐N value was calculated based on the consumption of hydrochloric acid.

### 2.5 Data analysis

Because the bioimpedance characteristics always carried redundant data, principal component analysis (PCA) was performed to simplify the variables. For qualitative analysis of the results, linear discriminant analysis (LDA), support vector machine (SVM), and back propagation artificial neural network (BPANN) were used. A partial least squares (PLS) model was employed to predict the TVB‐N value measured in the salmon samples from the biological impedance data (modulus and phase). For this analysis, each sample was divided into two groups to establish models. One group of salmon samples (training set) composed of two‐thirds of the salmon sample measurements was used to establish the regression model. The remaining group (test set) was used as the testing the model.

## 3 RESULTS AND DISCUSSION

### 3.1 Identification of Atlantic salmon and rainbow trout based on impedance characteristics

The impedance characteristics of biological tissues are well correlated with properties such as texture and fat content (Ando et al., 2016; Traffano‐Schiffo, Castro‐Giraldez, Colom, & Fito, 2018; Zavadlav et al., 2016; Zollinger, Farrow, Lawrence, & Latman, 2010). Different kinds of meat have different quality characteristics, such as different fiber thickness, texture, and fat content. Hence, we assumed that Atlantic salmon (A S) and rainbow trout (R T) would have different impedance characteristics. In this study, we analyzed and compared the electrical impedance spectra of A S and R T to distinguish the two different meat species.

**Figure 1:**
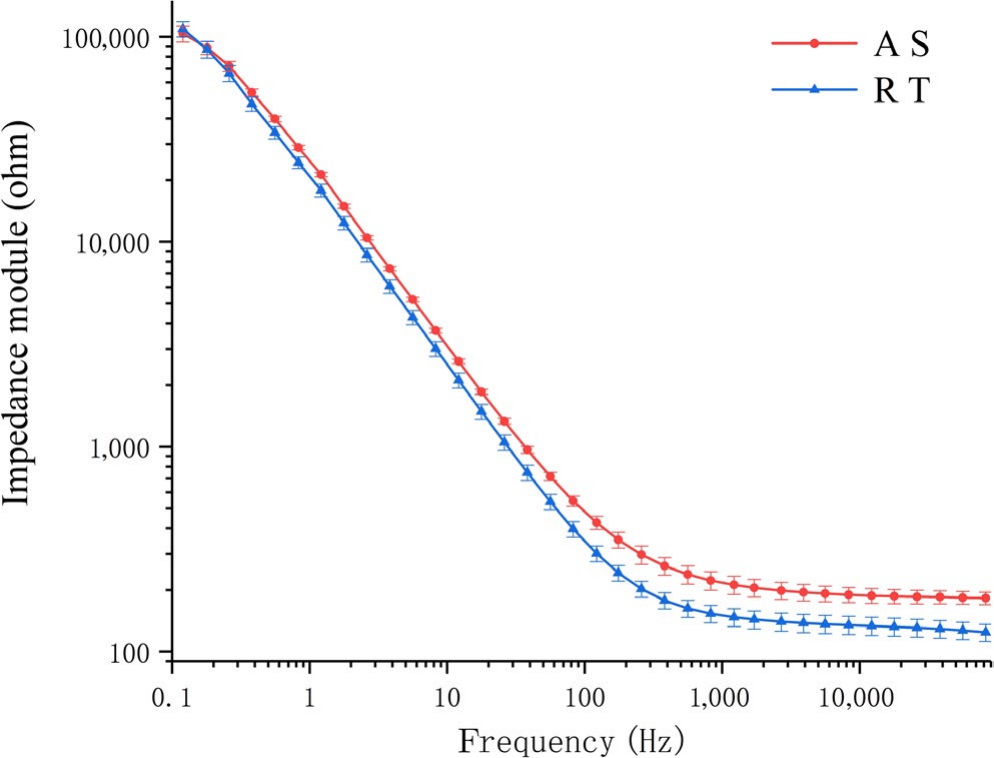
Impedance module curve of bode plots of Atlantic salmon and rainbow trout

**Figure 2:**
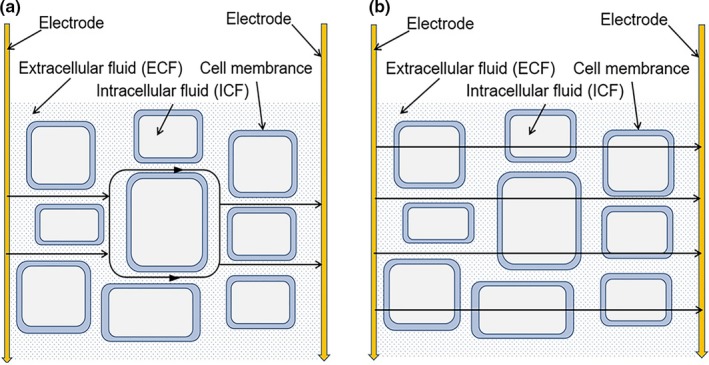
Current path in biological tissue. (a) Current path in biological tissue (low‐frequency current). (b) Current path in biological tissue (high‐frequency current)

Figure 1 is impedance module curve of bode plots of A S and R T. The horizontal axis represents frequency of excitation current signals, and the vertical axis represents the magnitude of biological impedance module. Figure 2 shows that the impedance module decreased with an increase in frequency; this effect can be explained by the path of current in the biological tissue. The cell membrane acts as insulators at low frequencies, similar to the action of capacitors (Pérez‐Esteve et al., 2014). The intracellular fluid with conductive properties could be equivalent to resistors, and the current blocked by the cell membrane can pass only through extracellular fluid (Figure 2a). Conversely, the high‐frequency current can pass through the cell membrane, so that the extracellular and intracellular fluids are equivalent to an in series (Figure 2b), and the increase in conducting section acreage would lead to a decrease in impedance (Kyle et al., 2004). In addition, the impedance modulus of A S was obviously higher than that of R T in the high‐frequency band of Figure 1. Furthermore, the impedance difference between A S and R T was not obvious at low frequencies. This lack of difference occurred because a low‐frequency current cannot pass through the cell membrane, and the equivalent capacitance between the sample and the electrode increased rapidly with the decrease of in frequency, which means that the weight of the inherent electrical properties of the fish we really want decreases (Sun, Zhang, Zhang, Li, & Liang, 2017), resulting in an impedance value that does not reflect the complete properties of the tissue (Chevalier, Sequeira‐Munoz, Bail, Simpson, & Ghoul, 2000).

**Figure 3:**
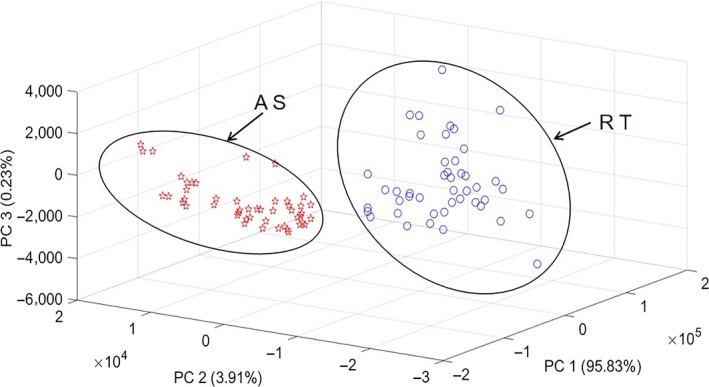
Score cluster plot of the top three principle components (PCs) for distinction between Atlantic salmon and rainbow trout

We first normalized the impedance modules and phase values of each fish fillet, and then placed them next to each other in a two‐block matrix. We used PCA to simplify 96 impedance measurements of the A S and R T samples (Figure 3). The first three PCs accounted for the information of the variables of 99.97%. As shown in Figure 3, there was a good distinction between the A S and R T groups in the score cluster plot. We further used LDA, BPANN, and SVM discriminant model to distinguish samples (see Table 1).

**Table 1:** Identification rate of salmon and rainbow trout by LDA, SVM, and BPANN Models

PCs	Identification rate of training set (%)			Identification rate of test set (%)		
	LDA	BPANN	SVM	LDA	BPANN	SVM
1	67.19	91.41	89.06	67.19	87.50	90.62
2	76.56	98.44	92.97	75.00	93.75	93.75
3	81.25	98.44	98.43	75.00	96.88	95.31
4	96.88	100	100	93.75	100	100
5	97.66	100	100	95.31	100	100
6	98.44	100	100	98.44	100	100
7	99.22	100	100	100	100	100
8	100	100	100	100	100	100

As shown in Table 1, the three models had relatively high recognition rates. When the number of principal components (PCs) was four, the discrimination accuracy for the two kinds of fish using the SVM or BPANN was nearly 100% in both the prediction set and calibration set. When the number of PCs was eight, the identification results of LDA also achieved 100%. The results showed that our method based on impedance characteristics combined with chemometrics was effective in distinguishing between A S and R T.

### 3.2 Identification of chilled salmon and frozen‐thawed salmon based on impedance characteristics

**Figure 4:**
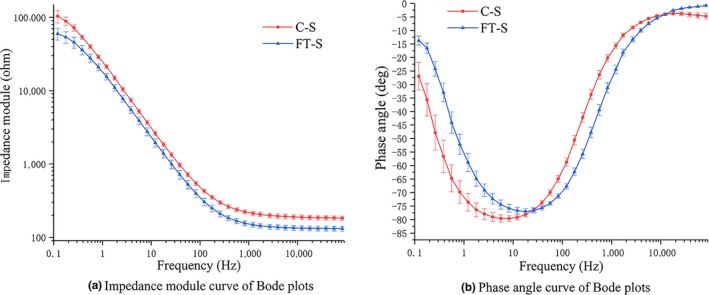
Bode plots of chilled/frozen‐thawed salmon

Freezing can increase the shelf life of meat, but freezing can destroy the cell membranes of biological tissues. The destruction of cell membranes causes release of nutrients and electrolytes, which then increases the conductivity and decreases the impedance modulus (Chen et al., 2017). Figure 4a,b shows the changes in impedance module and phase angle of frozen‐thawed samples (FT‐S) and chilled samples (C‐S). As shown in Figure 4a, the impedance modulus of FT‐S was lower than that of C‐S at all frequencies.

**Figure 5:**
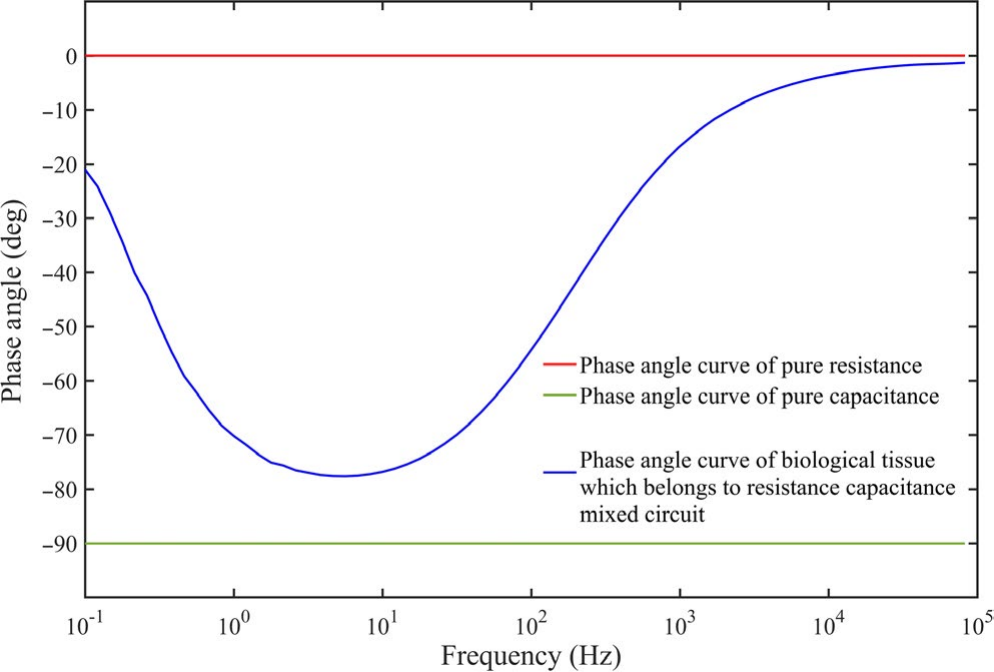
Typical phase angle curves of pure resistance, pure capacitance, and the resistance–capacitance mixed circuit

The cell membrane acts as a capacitor at low frequencies (Pliquett, 2010). Thus, the more intact the membrane, the greater the capacitive effect of a biological tissue. As shown in Figure 5, the horizontal line intersecting with the *y*‐axis at 0 is the phase curve of pure resistance, and the horizontal line intersecting the *y*‐axis at −90 is the phase curve of pure capacitance (Sun et al., 2018). The curve intersecting the *y*‐axis between 0 and −90 is the phase angle curve of the resistance–capacitance mixed circuit. Therefore, the closer the phase value is to −90°, the greater will be the capacitive effect. The membranes of C‐S were relatively intact (Leygonie, Britz, & Hoffman, 2012), but in FT‐S, most of cell membranes were damaged. This damage reduced the capacitance of the tissue, thereby reducing the value of the impedance phase (Ballin & Lametsch 2008). As shown in Figure 4b, the minimum phase angle of the C‐S could be lower than that of the FT‐S, and it could be as close as −90°.

**Figure 6:**
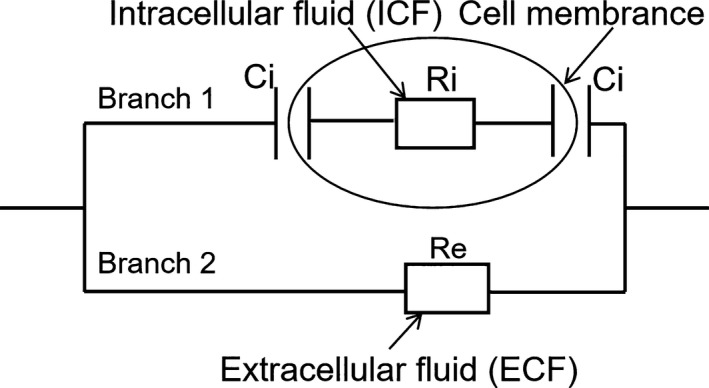
Equivalent circuit model of the organism. Re resistance of extracellular fluids, Ri resistance of intracellular fluids, Ci capacitor of cell membrane

In addition, the phase value increases when frequency is <10 Hz. These findings agreed with results obtained by Fuentes et al. (2013) for sea bream. We analyzed the mechanism of change in electrical properties by using the equivalent circuit model. The electric circuit in Figure 6 is the three‐component model, which is widely used in biological impedance analysis (Kyle et al., 2004). Two parallel branches represent the electrical characteristics of biological tissue. Branch 1 consists of intracellular fluids, which is equivalent to the resistance (Ri), and cell membranes, which is equivalent to the capacitance (Ci). Branch 2 consists of a pure resistance (Re) equivalent to extracellular fluids. When the frequency of alternating current becomes low, the equivalent capacitance of a cell membrane will become so large that Branch 2 (Figure 6) will be short‐circuited. Therefore, biological tissue shows a tendency toward pure resistance at this point, and the phase value of biological tissue increases.

**Figure 7:**
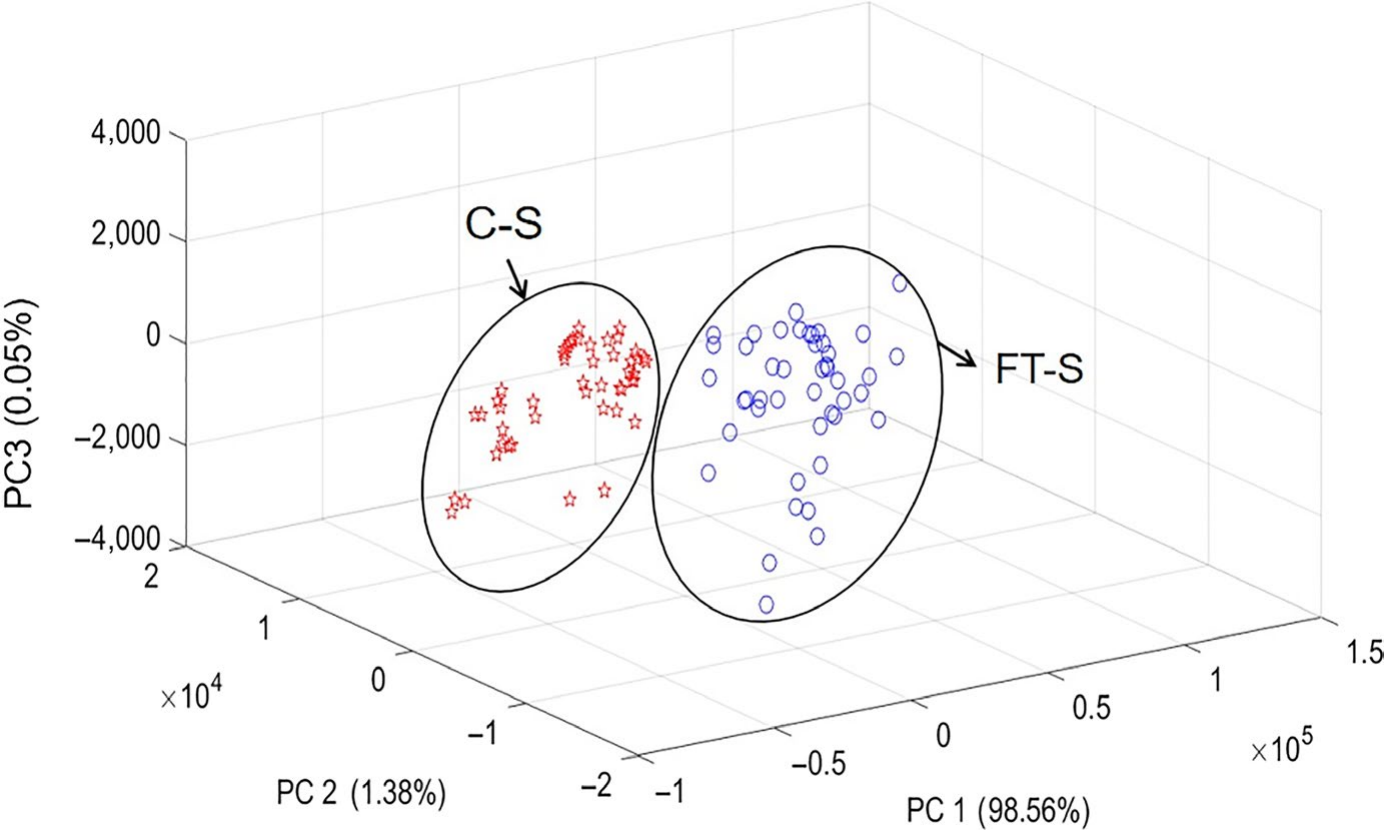
Score cluster plot of the top three principle components (PCs) for distinction between chilled and frozen‐thawed salmon

We first normalized the impedance modules and phase values of each fish fillet and then placed them next to each other in a two‐block matrix. We used PCA to simplify 96 C‐S and FT‐S impedance measurements. As shown in Figure 7, the first three PCs accounted for all variations of 99.99%. In addition, there was a clear distinction between the two types of samples in the space of the first three PCs. We further distinguished the two groups of samples by LDA, BPANN, and SVM discriminant model. All identification results of three discriminant models achieved 100%. The results showed that the combination of impedance characteristics and chemometrics can effectively distinguish C‐S from FT‐S.

### 3.3 Partial least squares regression for establishing relationships between impedance characteristic and control freshness

As the time since death increased, most muscle groups became separated from fibers, and the muscle structure was damaged (Caballero et al., 2009). Generally, when degradation of muscle, micro‐organisms, or enzymes release metabolites from cells, the conductivity of muscle tissue increases (Marshall & Wiese‐Lehigh 1997, Yang et al., 2013). This increase in conductivity provides an ability of impedance characteristics to predict changes in muscle integrity during storage and a feasibility of impedance characteristics to measure freshness.

**Figure 8:**
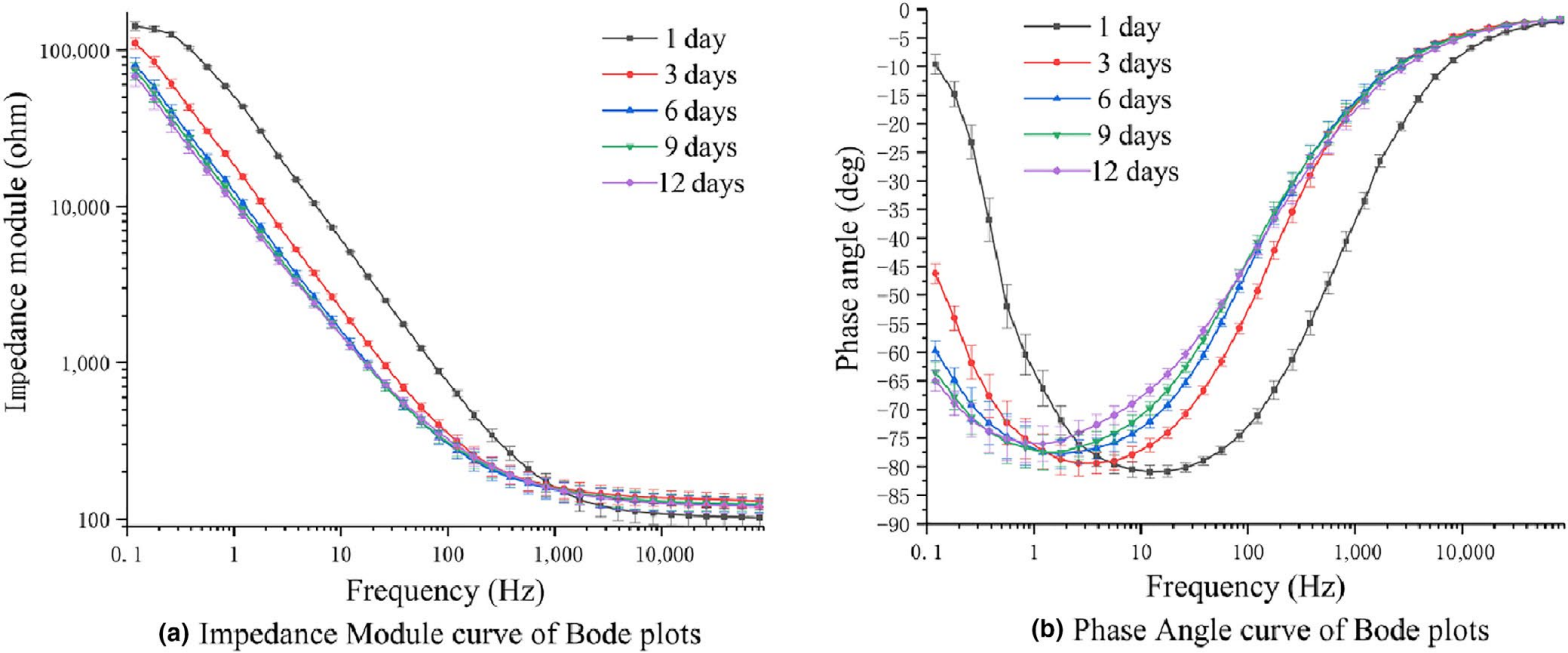
Bode plots of salmon stored for different days

Figure 8 shows the impedance module curve and the phase angle curve of Bode plots of salmon stored for different days. The impedance module of samples at lower frequencies decreased with the increasing storage days. In addition, the lowest point of phase angle curve increased gradually with the increase of storage days.

**Figure 9:**
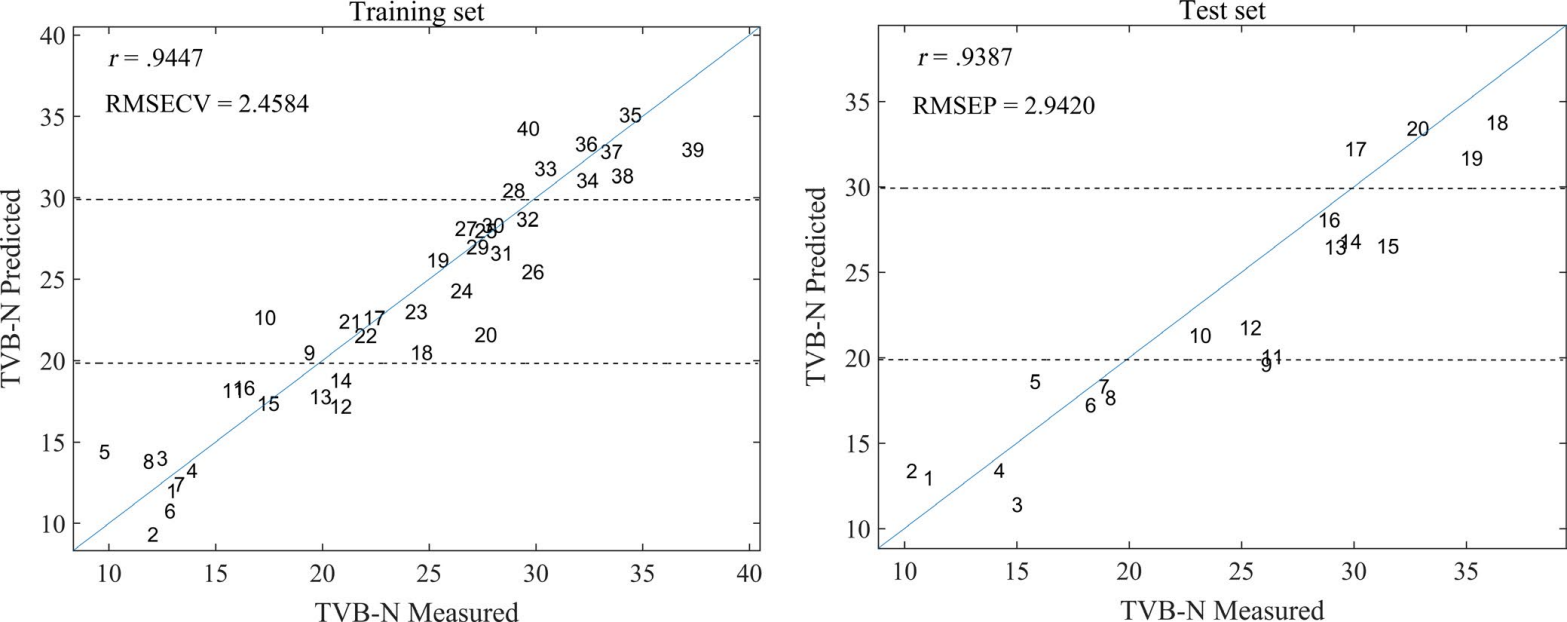
Correlation between measured and predicted values of TVB‐N

Total volatile base nitrogen is an important parameter to determine fish freshness. Thus, the freshness of fish fillets was represented by the content of TVB‐N in this paper. And, we applied PLS to establish a quantitative prediction model of bioimpedance spectroscopy and TVB‐N. The TVB‐N values for freshly caught marine fish are mostly 5–20 mg/100 g; 30 mg/100 g is generally considered an acceptable limit (EEC, 1995). As shown in Figure 9, in the first 3 days, almost all salmon samples were below the TVB‐N limit of 20 mg/100 g, belonging to the first‐class fresh range. On the 6th and 9th days, most salmon samples were in the range of 20–30 mg/100 g, belonging to the sub‐freshness level, and one sample exceeded the acceptable limit. On the 12th day, all salmon samples exceeded the acceptable limit and we considered them as rotten meat. The minimum RMSECV and RMSEP values of the model were 2.4584 mg/100 g and 2.9420 mg/100 g, and the correlation coefficients of training set and test set were 0.9447 and 0.9387. In summary, the impedance characteristics of biological tissues correlated well with their freshness.

## 4 CONCLUSIONS

We have used biological impedance properties combined with chemometrics to detect common salmon quality problems in the market. Our study included the identification of Atlantic salmon and rainbow trout, identification between chilled and frozen‐thawed salmon, and the prediction of salmon freshness.

We found obvious differences in the impedance spectra of Atlantic salmon versus rainbow trout and chilled versus frozen‐thawed salmon. LDA, BPANN, and SVM based on impedance characteristics achieved 100% recognition accuracy in training set and prediction set.

By using PLS to relate the bioimpedance data with the TVB‐N values of measured samples, we established quantitative relationships that showed good performance for predicting TVB‐N. In sum, impedance properties could accurately predict salmon freshness.

Finally, our study demonstrated that Atlantic salmon/rainbow trout, chilled/frozen‐thawed salmon, and fresh/stale salmon could be distinguished quickly and nondestructively to create multi‐quality indices for assessment of Atlantic salmon quality.

## CONFLICT OF INTEREST

The authors declare that they have no conflict of interest.

## ETHICAL APPROVAL

All experiments involving animals were conducted according to the principles of the Chinese Academy of Agricultural Sciences Animal Care and Use Committee (Beijing, China), which approved the study protocols.

## References

[fsn31362-bib-0001] Ali, S. , Zhang, W. , Rajput, N. , Khan, M. A. , Li, C. B. , & Zhou, G. H. (2015). Effect of multiple freeze–thaw cycles on the quality of chicken breast meat. Food Chemistry, 173, 808–814. 25466093 10.1016/j.foodchem.2014.09.095

[fsn31362-bib-0002] Ando, Y. , Maeda, Y. , Mizutani, K. , Wakatsuki, N. , Hagiwara, S. , & Nabetani, H. (2016). Effect of air‐dehydration pretreatment before freezing on the electrical impedance characteristics and texture of carrots. Journal of Food Engineering, 169, 114–121.

[fsn31362-bib-0003] Aursand, I. G. , Erikson, U. , & Veliyulin, E. (2010). Water properties and salt uptake in Atlantic salmon fillets as affected by ante‐mortem stress, rigor mortis, and brine salting: A low‐field 1H NMR and 1H/23Na MRI study. Food Chemistry, 120(2), 482–489.

[fsn31362-bib-0004] Ballin, N. Z. , & Lametsch, R. (2008). Analytical methods for authentication of fresh vs. thawed meat–A review. Meat Science, 80(2), 151–158. 22063317 10.1016/j.meatsci.2007.12.024

[fsn31362-bib-0005] Bosworth, B. G. , & Wolters, W. R. (2001). Evaluation of bioelectric impedance to predict carcass yield, carcass composition, and fillet composition in farm‐raised catfish. Journal of the World Aquaculture Society, 32(1), 72–78.

[fsn31362-bib-0006] Caballero, M. J. , Betancor, M. , Escrig, J. C. , Montero, D. , Espinosa de los Monteros, A. , Castro, P. , … Izquierdo, M. (2009). Post mortem changes produced in the muscle of sea bream (*Sparus aurata*) during ice storage. Aquaculture, 291(3–4), 210–216. 10.1016/j.aquaculture.2009.03.032

[fsn31362-bib-0007] Chen, T. H. , Zhu, Y. P. , Han, M. Y. , Wang, P. , Wei, R. , Xu, X. L. , & Zhou, G. H. (2017). Classification of chicken muscle with different freeze–thaw cycles using impedance and physicochemical properties. Journal of Food Engineering, 196, 94–100.

[fsn31362-bib-0008] Chevalier, D. , Sequeira‐Munoz, A. , Le Bail, A. , Simpson, B. K. , & Ghoul, M. (2000). Effect of freezing conditions and storage on ice crystal and drip volume in turbot (*Scophthalmus maximus*): Evaluation of pressure shift freezing vs. air‐blast freezing. Innovative Food Science & Emerging Technologies, 1(3), 193–201.

[fsn31362-bib-0009] Cox, M. K. , & Hartman, K. J. (2005). Nonlethal estimation of proximate composition in fish. Canadian Journal of Fisheries and Aquatic Science, 62(2), 269–275.

[fsn31362-bib-0010] EEC (1995). Total volatile basic nitrogen (TVB‐N) limit values for certain categories of fishery products and specifying the analysis methods to be used. Commission Decision 95/149/EEC of 8 March 1995. Official Journal of European Union, L97, 84–87.

[fsn31362-bib-0011] Fuentes, A. , Masot, R. , Fernández‐Segovia, I. , Ruiz‐Rico, M. , Alcañiz, M. , & Barat, J. M. (2013). Differentiation between fresh and frozen‐thawed sea bream (*Sparus aurata*) using impedance spectroscopy techniques. Innovative Food Science and Emerging Technologies, 19, 210–217.

[fsn31362-bib-0012] Gheorghiu, E. U. G. E. N. (1993). The dielectric behaviour of a biological cell suspension. Romanian Journal of Physics, 38, 113–113.

[fsn31362-bib-0013] Johnston, I. A. , Li, X. , Vieira, V. L. , Nickell, D. , Dingwall, A. , Alderson, R. , … Bickerdike, R. (2006). Muscle and flesh quality traits in wild and farmed Atlantic salmon. Aquaculture, 256(1–4), 323–336.

[fsn31362-bib-0014] Kyle, U. G. , Bosaeus, I. , De Lorenzo, A. D. , Deurenberg, P. , Elia, M. , Gómez, J. M. , … Scharfetter, H. (2004). Bioelectrical impedance analysis—Part I: Review of principles and methods. Clinical Nutrition, 23(5), 1226–1243. 15380917 10.1016/j.clnu.2004.06.004

[fsn31362-bib-0015] Leygonie, C. , Britz, T. J. , & Hoffman, L. C. (2012). Impact of freezing and thawing on the quality of meat. Meat Science, 91(2), 93–98. 22326063 10.1016/j.meatsci.2012.01.013

[fsn31362-bib-0016] Marshall, D. L. , & Wiese‐Lehigh, P. L. (1997). Comparison of impedance, microbial, sensory, and pH methods to determine shrimp quality. Journal of Aquatic Food Product Technology, 6(2), 17–31. 10.1300/J030v06n02_03

[fsn31362-bib-0017] Pérez‐Esteve, E. , Fuentes, A. , Grau, R. , Fernández‐Segovia, I. , Masot, R. , Alcañiz, M. , & Barat, J. M. (2014). Use of impedance spectroscopy for predicting freshness of sea bream (*Sparus aurata*). Food Control, 35(1), 360–365.

[fsn31362-bib-0018] Pliquett, U. (2010). Bioimpedance: A review for food processing. Food Engineering Reviews, 2(2), 74–94.

[fsn31362-bib-0019] Quevedo, R. , & Aguilera, J. M. (2010). Computer vision and stereoscopy for estimating firmness in the salmon (*Salmon salar*) fillets. Food and Bioprocess Technology, 3(4), 561–567.

[fsn31362-bib-0020] Quevedo, R. A. , Aguilera, J. M. , & Pedreschi, F. (2010). Color of salmon fillets by computer vision and sensory panel. Food and Bioprocess Technology, 3(5), 637–643.

[fsn31362-bib-0021] Rizo, A. , Fuentes, A. , Fernández‐Segovia, I. , Masot, R. , Alcañiz, M. , & Barat, J. M. (2013). Development of a new salmon salting–smoking method and process monitoring by impedance spectroscopy. LWT‐Food Science and Technology, 51(1), 218–224.

[fsn31362-bib-0022] Rodríguez, A. , Cruz, J. M. , Paseiro‐Losada, P. , & Aubourg, S. P. (2012). Effect of a polyphenol–vacuum packaging on lipid deterioration during an 18‐month frozen storage of Coho Salmon (*Oncorhynchus kisutch*). Food and Bioprocess Technology, 5(6), 2602–2611.

[fsn31362-bib-0023] Sallam, K. I. (2007). Chemical, sensory and shelf life evaluation of sliced salmon treated with salts of organic acids. Food Chemistry, 101(2), 592–600. 17245440 10.1016/j.foodchem.2006.02.019 PMC1780258

[fsn31362-bib-0024] Skipnes, D. , Johnsen, S. O. , Skåra, T. , Sivertsvik, M. , & Lekang, O. (2011). Optimization of heat processing of farmed Atlantic cod (*Gadus morhua*) muscle with respect to cook loss, water holding capacity, color, and texture. Journal of Aquatic Food Product Technology, 20(3), 331–340.

[fsn31362-bib-0025] Sun, J. , Zhang, R. , Zhang, Y. , Li, G. , & Liang, Q. (2017). Estimating freshness of carp based on EIS morphological characteristic. Journal of Food Engineering, 193, 58–67.

[fsn31362-bib-0026] Sun, J. , Zhang, R. , Zhang, Y. , Liang, Q. , Li, G. , Yang, N. , … Guo, J. (2018). Classifying fish freshness according to the relationship between EIS parameters and spoilage stages. Journal of Food Engineering, 219, 101–110.

[fsn31362-bib-0027] Tito, N. B. , Rodemann, T. , & Powell, S. M. (2012). Use of near infrared spectroscopy to predict microbial numbers on Atlantic salmon. Food Microbiology, 32(2), 431–436. 22986211 10.1016/j.fm.2012.07.009

[fsn31362-bib-0028] Traffano‐Schiffo, M. V. , Castro‐Giraldez, M. , Colom, R. J. , & Fito, P. J. (2018). Innovative photonic system in radiofrequency and microwave range to determine chicken meat quality. Journal of Food Engineering, 239, 1–7.

[fsn31362-bib-0029] Willis, J. , & Hobday, A. J. (2008). Application of bioelectrical impedance analysis as a method for estimating composition and metabolic condition of southern bluefin tuna (*Thunnus maccoyii*) during conventional tagging. Fisheries Research, 93(1–2), 64–71.

[fsn31362-bib-0030] Yang, Y. , Wang, Z. Y. , Ding, Q. , Huang, L. , Wang, C. , & Zhu, D. Z. (2013). Moisture content prediction of porcine meat by bioelectrical impedance spectroscopy. Mathematical and Computer Modelling, 58(3–4), 819–825.

[fsn31362-bib-0031] Zavadlav, S. , Janči, T. , Lacković, I. , Karlović, S. , Rogulj, I. , & Vidaček, S. (2016). Assessment of storage shelf life of European squid (cephalopod: Loliginidae, *Loligo vulgaris*) by bioelectrical impedance measurements. Journal of Food Engineering, 184, 44–52.

[fsn31362-bib-0032] Zhang, L. , Shen, H. , & Luo, Y. (2011). A nondestructive method for estimating freshness of freshwater fish. European Food Research and Technology, 232(6), 979–984.

[fsn31362-bib-0033] Zollinger, B. L. , Farrow, R. L. , Lawrence, T. E. , & Latman, N. S. (2010). Prediction of beef carcass salable yield and trimmable fat using bioelectrical impedance analysis. Meat Science, 84(3), 449–454. 20374809 10.1016/j.meatsci.2009.09.015

